# Overexpression of cell cycle regulator CDCA3 promotes oral cancer progression by enhancing cell proliferation with prevention of G1 phase arrest

**DOI:** 10.1186/1471-2407-12-321

**Published:** 2012-07-28

**Authors:** Fumihiko Uchida, Katsuhiro Uzawa, Atsushi Kasamatsu, Hiroaki Takatori, Yosuke Sakamoto, Katsunori Ogawara, Masashi Shiiba, Hideki Tanzawa, Hiroki Bukawa

**Affiliations:** 1 Department of Oral and Maxillofacial Surgery, Clinical Sciences, Graduate School of Comprehensive Human Sciences, University of Tsukuba, 1-1-1 Tennodai, Tsukuba, Ibaraki, 305-8575, Japan; 2 Department of Clinical Molecular Biology, Graduate School of Medicine, Chiba University, 1-8-1 Inohana, Chuo-ku, Chiba 260-8670, Japan; 3 Division of Dentistry and Oral-Maxillofacial Surgery, Chiba University Hospital, 1-8-1 Inohana, Chuo-ku, Chiba 260-8670, Japan; 4 Department of Molecular Genetics, Graduate School of Medicine, Chiba University, 1-8-1 Inohana, Chuo-ku, Chiba 260-8670, Japan; 5 Department of Oral and Maxillofacial Surgery, Faculty of Medicine, University of Tsukuba, 1-1-1 Tennodai, Tsukuba, Ibaraki 305-8575, Japan

**Keywords:** Cell division cycle associated 3, Oral squamous cell carcinoma, Proliferation, Cell-cycle arrest, Cyclin-dependent kinase inhibitors

## Abstract

**Background:**

Cell division cycle associated 3 (CDCA3), part of the Skp1-cullin-F-box (SCF) ubiquitin ligase, refers to a trigger of mitotic entry and mediates destruction of the mitosis inhibitory kinase. Little is known about the relevance of CDCA3 to human malignancy including oral squamous cell carcinoma (OSCC). We aimed to characterize the expression state and function of CDCA3 in OSCC.

**Methods:**

We evaluated CDCA3 mRNA and protein expression in both OSCC-derived cell lines and primary OSCCs and performed functional analyses of CDCA3 in OSCC-derived cells using the shRNA system.

**Results:**

The CDCA3 expression at both the mRNA and protein levels was frequently up-regulated in all cell lines examined and primary tumors (mRNA, 51/69, 74 %; protein, 79/95, 83 %) compared to normal controls (*p* < 0.001). In contrast, no significant level of CDCA3 protein expression was seen in oral premalignant lesions (OPLs) (n *=* 20) compared with the expression in OSCCs. Among the clinical variables analyzed, the CDCA3 expression status was closely related to tumor size (*p* < 0.05). In addition, suppression of CDCA3 expression with shRNA significantly (*p* < 0.05) inhibited cellular proliferation compared with the control cells by arresting cell-cycle progression at the G1 phase. Further, there was up-regulation of the cyclin-dependent kinase inhibitors (p21^Cip1^, p27^Kip1^, p15^INK4B^, and p16^INK4A^) in the knockdown cells.

**Conclusion:**

The current results showed that overexpression of CDCA3 occurs frequently during oral carcinogenesis and this overexpression might be associated closely with progression of OSCCs by preventing the arrest of cell-cycle progression at the G1 phase via decreased expression of the cyclin-dependent kinase inhibitors.

## Background

Rapid cellular growth and division are common features in all malignant cells including oral squamous carcinoma [1]. It is well documented that inappropriate expression of cell-cycle regulatory proteins can contribute to human tumorigenesis [2]. Numerous studies have reported the relation between carcinogenesis and the cell-cycle-related gene [3-6]. In particular, recent studies have suggested that deregulation of Skp1-cullin-F-box (SCF) control of the G1/S phase targets also might contribute to human tumorigenesis [3,7-9].

Our previous microarray analysis showed that *CDCA3*, referred to as a trigger of mitotic entry, mediates destruction of mitosis and the inhibitory kinase via the E3 ligase, SCF [10-14] and was one of the up-regulated genes in the oral squamous cell carcinoma (OSCC)-derived cells [15]. The ubiquitin-proteasome system is one of the critical mechanisms controlling protein turnover and thus maintains cellular protein homeostasis [16]. Although protein ubiquitination is catalyzed by a highly ordered enzymatic cascade, including ubiquitin-activating enzyme E1s, ubiquitin-conjugating enzyme E2s, and ubiquitin ligase E3s, the last of which primarily determine the substrate specificity [16,17]. The SCF, E3 ubiquitin ligases, consisting of Skp1, cullins/cdc53, F-box proteins, and the RING domain containing protein regulator of cullins-1 (ROC1)/ring box protein-1, are crucial to the regulation of numerous cellular processes under both physiologic and pathologic conditions as part of the ubiquitin-proteosome system [18]. By promoting degradation of many short-lived proteins, including cell cycle regulators, transcription factors and signal transducers, SCF E3 ligases regulate many biological processes [19]. CDCA3 is a protein that contains an F box motif and bind to Skp1 and cullin, a component of SCF. Since the F-box protein determines the specificity of SCF ligases, it represents a target that could provide the greatest potential selectivity [20]. As a critical cell-cycle regulator, p27 ^Kip1^ arrests cell division and inhibits G_1_/S transition, and the cellular p27^Kip1^ levels are modulated largely through the ubiquitin-proteasome pathway [21]. Thus, progression of the cell cycle can be regulated by modulating the quantities of cell-cycle regulators through ubiquitination by the SCF complex [1]. However, the expression state and function of CDCA3 in OSCCs are not fully characterized.

The current study shows the results of a comprehensive analysis of aberrant expression of CDCA3 in OSCCs that are clinically and functionally linked to tumor progression.

## Methods

### Cell culture

HSC-2, HSC-3, HSC-4, and Ca9-22 cell lines, derived from human OSCCs, were purchased from the Human Science Research Resources Bank, Osaka, Japan. H1 and Sa3 were kindly provided by Dr. S. Fujita of Wakayama Medical University, Wakayama, Japan. HNOKs were used as a normal control [22,23]. All cells were grown in Dulbecco’s modified Eagle medium (DMEM) F-12 HAM (Sigma Aldrich, St. Louis, MO) supplemented with 10% fetal bovine serum (FBS) (Sigma) and 50 units/ml penicillin and streptomycin (Sigma).

### Tissue specimens

Primary OSCC samples and corresponding normal oral epithelial tissues were obtained at the time of surgeries performed at Chiba University Hospital. All patients provided informed consent for the study protocol, which was approved by the institutional review board of Chiba University. The tissues were divided into two parts, one of which was frozen immediately and stored at −80°C until RNA isolation, and the second was fixed in 20% buffered formaldehyde solution for pathologic diagnosis and IHC. The Department of Pathology, Chiba University Hospital, performed the histopathologic diagnosis of each tissue according to the World Health Organization criteria. Clinicopathological staging was determined according to the tumor-node-metastases classification of the International Union against Cancer. All OSCC samples were confirmed histologically and checked to ensure the presence of tumor in greater than 90% of specimens.

### Preparation of cDNA

Total RNA was isolated using Trizol Reagent (Invitrogen, Carlsbad, CA) according to the manufacturer’s instructions. cDNA was generated from 5 μg of total RNA using Ready-To-Go You-Prime First-Strand Beads (GE Healthcare, Buckinghamshire, UK) and oligo (dT) primer (Sigma Genosys, Ishikari, Japan), according to the manufacturer’s instructions.

### mRNA expression analysis

qRT-PCR was performed to evaluate the expression levels of *CDCA3* and *Wee1* mRNA in OSCC-derived cell lines and HNOKs. We also evaluated the mRNA levels in primary OSCCs and paired specimens of normal oral tissues obtained from 69 patients. qRT-PCR was performed using LightCycler 480 apparatus (Roche Diagnostics GmbH, Mannheim, Germany). Primers were designed using the ProbeFinder qPCR assay design software, which is freely accessible at http://www.universalprobelibrary.com. The sequences of the gene-specific primers were as follows: *CDCA3* forward 5’-TGGTATTGCACGGACACCTA-3’ and reverse 5’-TGTTTCACCAGTGGGCTTG-3’; *Wee1* forward 5’-TTTGGTTCACATGGATATAAAACCT-3’ and reverse 5’-CCCAATCATCTTCGTCTCCT-3’. The PCR reactions were carried out in a final volume of 20 μl of a reaction mixture comprised of 10 μl of LightCycler 480 Probes Master (Roche), 0.2 μl of universal probe (Roche), and 4 μM of the primers, according to the manufacturer’s instructions. The reaction mixture was loaded onto the PCR plate and subjected to an initial denaturation at 95°C for 10 min, followed by 45 rounds of amplification at 95°C (10 sec) for denaturation, 60°C (30 sec) for annealing, and 72°C (1 sec) for extension, followed by a cooling step at 50°C for 30 seconds. The transcript amounts for the *CDCA3* and *Wee1* genes were estimated from the respective standard curves and normalized to the *glyceraldehyde-3-phosphate dehydrogenase* (*GAPDH*) forward 5’-AGCCACATCGCTCAGACAC-3’ and reverse 5’-GCCCAATACGACCAAATCC-3’ transcript amount determined in corresponding samples.

### Protein expression analysis

The cells were washed twice with cold phosphate buffered saline (PBS) and centrifuged briefly. The cell pellets were incubated at 4°C for 30 min in a lysis buffer (7 M urea, 2 M thiourea, 4% w/v CHAPS, and 10 mM Tris pH 7.4) with a proteinase inhibitor cocktail (Roche). The protein concentration was measured using the Bradford reagent (Bio-Rad, Richmond, CA). Protein extracts were electrophoresed on 4% to 12% Bis-Tris gel, transferred to nitrocellulose membranes (Invitrogen), and blocked for 1 hr at room temperature with Blocking One (Nacalai Tesque, Inc., Kyoto, Japan). The membranes were washed three times with 0.1 % Tween-20 in Tris-buffered saline and incubated with antibody for CDCA3 (Abcam, Cambridge, UK), p21^Cip1^, p27^Kip1^, p15^INK4B^, p16^INK4A^, CDK4, CDK6, Cyclin D1 (Cell Signaling Technology, Danvers, MA), and Cyclin E (Santa Cruz Biotechnology, Santa Cruz, CA) overnight at 4°C and α-tubulin (Santa Cruz Biotechnology) 1 hr at room temperature. The membranes were washed again and incubated with a anti-rabbit or anti-mouse IgG (H + L) horseradish peroxidase conjugate (Promega, Madison, WI) as a secondary antibody for 1 hr at room temperature. Finally, the membranes were detected using SuperSignal West Pico Chemiluminescent substrate (Thermo, Rockford, IL), and immunoblotting was visualized by exposing the membranes to ATTO Light-Capture II (Tokyo, Japan). Signal intensities were quantitated using the CS Analyzer version 3.0 software (ATTO).

### IHC

IHC of 4-μm sections of paraffin-embedded specimens was performed using rabbit anti-CDCA3 polyclonal antibody (Abcam). Briefly, after deparaffinization and hydration, the endogeneous peroxidase activity was quenched by a 30 min incubation in a mixture of 0.3% hydrogen peroxide solution in 100% methanol, after which the sections were blocked for 2 hr at room temperature with 1.5% blocking serum (Santa Cruz Biotechnology) in PBS before reaction overnight with anti-CDCA3 antibody (1:100 dilution) at 4°C in a moist chamber. Upon incubation with the primary antibody, the specimens were washed three times in PBS and treated with Envision reagent (DAKO, Carpinteria, CA) followed by color development in 3,3’-diaminobenzidine tetrahydrochloride (DAKO). The slides then were lightly counterstained with hematoxylin, dehydrated with ethanol, cleaned with xylene, and mounted. Non-specific binding of an antibody to proteins other than the antigen sometimes occurred. To avoid non-specific binding, an immunizing peptide blocking experiment was performed. As a negative control, triplicate sections were immunostained without exposure to primary antibodies, which confirmed the staining specificity. To quantify the status of the CDCA3 protein expression in those components, we used an IHC scoring system described previously [22-26]. This IHC scoring system was established for quantitative evaluation of IHC staining. The stained cells were determined in at least five random fields at 400 × magnification in each section. We counted 300 cells per one field of vision. The staining intensity (1, weak; 2, moderate; 3, intense) and the number of positive cells in the field of vision then were multiplied to calculate the IHC score using the following formula: IHC score = 1 × (number of weakly stained cells in the field) + 2 × (number of moderately stained cells in the field) + 3 × (number of intensely stained cells in the field). Cases with a CDCA3 IHC score exceeding 94.7 (maximum score within + 3 standard deviation (SD) of the mean of normal tissues) were defined as CDCA3-positive because 100% of the distribution falls within ± 3 SD of the mean in normal tissues. Two independent pathologists, both masked to the patients’ clinical status, made these judgments.

### Stable transfection of CDCA3 shRNA

Stable transfection was performed at about 80% confluency in 24 well plates using Lipofectamine LTX and Plus Reagents (Invitrogen), according to the manufacturer’s instructions. Briefly, a total of 2 × 10^5^ cells were seeded into each well in DMEM F-12 HAM (Sigma) containing 10% FBS (Sigma) without antibiotics. shCDCA3 and mock (0.1 μg) (Santa Cruz Biotechnology) vectors were transfected into OSCC-derived cells (H1 and Sa3) with 0.5 μl of Plus Reagents and 1.25 μl of Lipofectamine LTX. After transfection, the cells were isolated by the culture medium containing 2 μg/mL puromycin (Invitrogen). After 3 to 4 weeks, resistant cell clones were picked and transferred to 6-well plates and gradually expanded to 10-cm dishes. At 90% confluence, qRT-PCR and Western blot analyses were performed to assess the efficiency of CDCA3 knockdown.

### Cellular growth

To evaluate the effect of CDCA3 knockdown on cellular proliferation, we analyzed cellular growth in shCDCA3- and mock-transfected cells. These transfectants were seeded in 6-well plates at a density of 1 × 10^4^ viable cells per well. The experiments were carried out for 168 hr, and the cells were counted every 24 hr. At the indicated time point, the cells were trypsinized and counted using a hemocytometer in triplicate samples.

### Cell-cycle analysis

To assess cell-cycle distribution of entire cell populations, the cells were harvested, washed with PBS, and probed with CycleTEST Plus DNA reagent kit (Becton-Dickinson, San Jose, CA), according to the manufacturer’s protocol. Briefly, the cells were centrifuged at 400 × g for 5 min. The cell pellets were resuspended with 250 μl of trypsin buffer, and incubated for 10 min at room temperature. We then added 200 μl of trypsin inhibitor and RNase buffer. Finally, the cells were labeled with 200 μl of propidium iodide stain solution. Flow cytometric determination of DNA content was analyzed by FACSCalibur (Becton-Dickinson). The fractions of the cells in the G0-G1, S, and G2-M phases were analyzed using Flow Jo software (Tree Star, Ashland, OR).

### Statistical analysis

Statistical significance was determined using Fisher’s exact test or the Mann-Whitney’s *U* test. *p* < 0.05 was considered significant. The data are expressed as the mean ± standard error of the mean (SEM).

## Results

### Evaluation of CDCA3 expression in OSCC-derived cell lines and primary OSCCs

**Figure 1:**
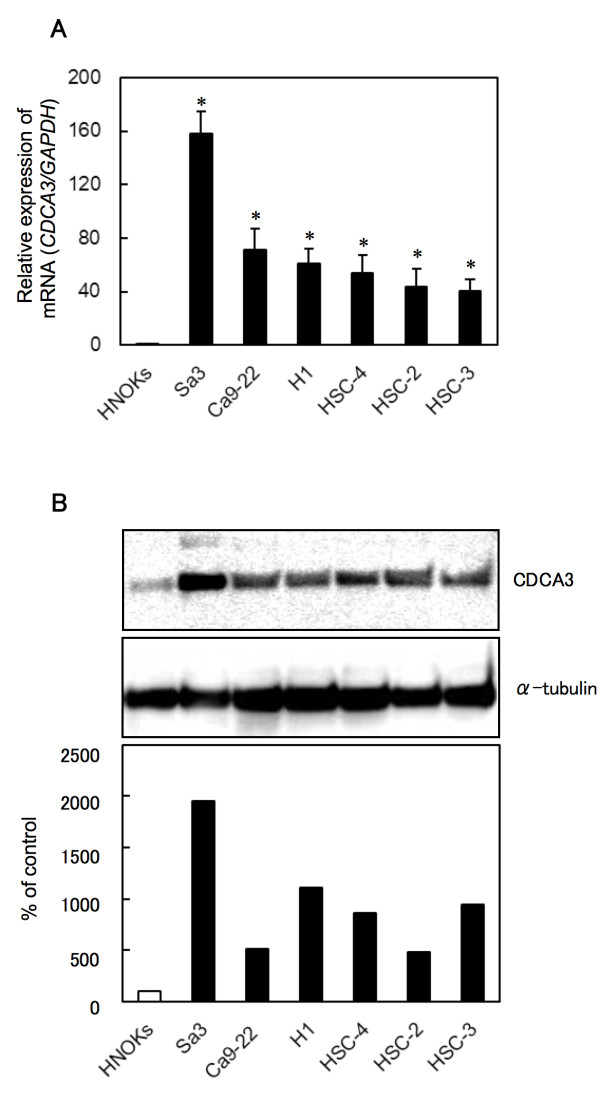
**Evaluation of CDCA3 expression in OSCC-derived cell lines. A**) Quantification of *CDCA3* mRNA expression in OSCC-derived cell lines by qRT-PCR analysis. Significant up-regulation of *CDCA3* mRNA is seen in six OSCC-derived cell lines compared with the HNOKs (*p < 0.05, Mann-Whitney’s *U* test). Data are expressed as the means ± SEM of triplicate results. **B**) A representative Western blot of CDCA3 protein in OSCC-derived cell lines and HNOKs shows that CDCA3 protein expression is up-regulated in OSCC-derived cell lines compared with HNOKs. Densitometric CDCA3 protein data are normalized to α-tubulin protein levels. The values are expressed as a percentage of the HNOKs.

**Figure 2:**
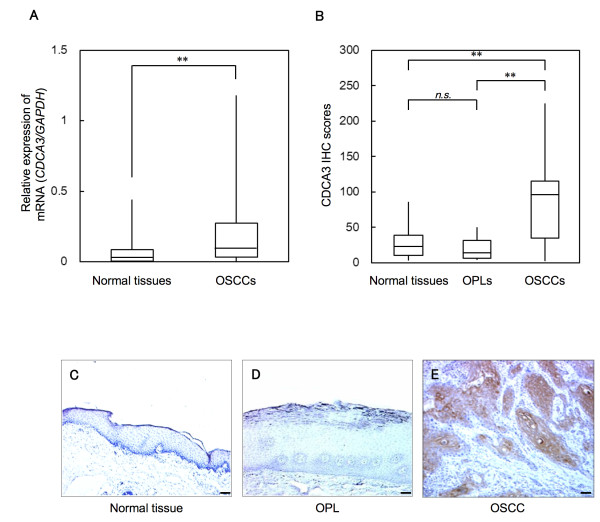
**Evaluation of CDCA3 expression in normal oral tissues, OPLs, and primary OSCCs. A**) qRT-PCR analysis shows that *CDCA3* mRNA expression is up-regulated in 51 (74 %) of 69 primary OSCCs compared with the matched normal oral tissues. The relative mRNA expression levels in the normal oral tissues and primary OSCCs range from 6.3 × 10^-5^ to 0.442 (median, 0.033) and 5.9 × 10^-5^ to 1.178 (median, 0.083), respectively. Significantly higher *CDCA3* mRNA expression is seen in primary OSCCs than matched normal oral tissues (**p < 0.001, Mann-Whitney’s *U* test). **B**) The status of CDCA3 protein expression in primary OSCCs and paired normal oral tissues from 95 patients and OPLs from 20 patients based on an IHC scoring system. The CDCA3 IHC scores of normal oral tissues, OPLs, and OSCCs range from 2.5 to 87.5 (median, 22.5), 2.5 to 50.0 (median, 15.0), and 2.5 to 225.0 (median, 95.0), respectively. The CDCA3 protein expression level in OSCCs is significantly higher (**p < 0.001, Mann-Whitney’s *U* test) than in normal oral tissues and OPLs. No significant (*p* = not significant [n.s.]) difference in protein expression is seen between OPLs and normal oral tissues. Representative IHC results of CDCA3 in normal oral tissues (**C**), OPLs (**D**) and primary OSCCs (**E**) (×100 magnification. Scale bars, 50 μm.). A strong CDCA3 immunoreaction is seen in OSCCs, whereas normal oral tissues and OPLs show almost negative immunostaining.

To investigate mRNA and protein expression of CDCA3 identified as a cancer-related gene in our previous microarray data [15], we performed real-time quantitative reverse transcriptase-polymerase chain reaction (qRT-PCR) and Western blot analyses using six OSCC-derived cell lines (HSC-2, HSC-3, HSC-4, Ca9-22, H1, and Sa3) and primary cultured human normal oral keratinocytes (HNOKs). *CDCA3* mRNA was significantly (*p < 0.05) up-regulated in all OSCC-derived cell lines compared with the HNOKs (Figure 1A). Figure 1B shows representative results of Western blot analysis. The molecular weight of CDCA3 was 29 kDa. A significant increase in CDCA3 protein expression was seen in all OSCC-derived cell lines compared with the HNOKs. Expression analysis indicated that both transcription and translation products of this molecule were highly expressed in OSCC-derived cell lines. We then measured the *CDCA3* mRNA expression levels in primary OSCCs and paired normal oral tissues from 69 patients (Figure 2A). Similar to the data from the OSCC-derived cell lines, qRT-PCR analysis showed that *CDCA3* mRNA expression was up-regulated in 51 (74%) of 69 primary OSCCs compared with the matched normal oral tissues. The relative mRNA expression levels in the normal oral tissues and primary OSCCs ranged from 6.3 × 10^-5^ to 0.442 (median, 0.033) and 5.9 × 10^-5^ to 1.178 (median, 0.083), respectively. When we analyzed the CDCA3 protein expression in primary OSCCs and paired normal oral tissues from 95 patients and oral premalignant lesions (OPLs) from 20 patients using the immunohistochemistry (IHC) scoring system, the CDCA3 IHC scores in the primary OSCCs, OPLs, and normal oral tissues ranged from 2.5 to 225.0 (median, 95.0), 2.5 to 50.0 (median, 15.0), and 2.5 to 87.5 (median, 22.5), respectively. The CDCA3 IHC score in primary OSCCs was significantly (**p < 0.001) higher than those in OPLs and normal oral tissues (Figure 2B); there was no significant difference in the IHC scores between the OPLs and normal oral tissues. Representative IHC results for CDCA3 protein in normal oral tissues, OPLs, and primary OSCCs are shown in Figure 2C, D, and E. Strong CDCA3 immunoreactions were detected in the cytoplasm in the OSCCs; the OPLs and normal oral tissues showed negative immunostaining. CDCA3 protein expression was up-regulated in 79 (83%) of 95 primary OSCCs compared with the matched normal oral tissues. The correlations between the clinicopathologic characteristics of the patients with OSCC and the status of the CDCA3 protein expression using the IHC scoring system are shown in Table 1. Among the clinical classifications, CDCA3-positive OSCCs were correlated significantly (*p < 0.05) with tumor size.

**Table 1:** Correlation between CDCA3 expression and clinical classification in OSCCs

		**Results of immunostaining**		
**Clinical classification**		**No. patients (%)**		
	**Total**	**CDCA3 negative**	**CDCA3 positive**	***p *value**
Age at surgery (years)				
<60	23	9 (39)	14 (61)	
≧60, <70	22	11 (50)	11 (50)	0.852
≧70	50	22 (44)	28 (56)	
Gender				
Male	58	28 (48)	30 (52)	0.317
Female	37	14 (38)	23 (62)	
T-primary tumor				
T1	6	3 (50)	3 (50)	0.020*
T2	56	30 (54)	26 (46)	
T3	15	5 (33)	10 (67)	
T4	18	4 (22)	14 (78)	
T1 + T2	62	33 (53)	29 (47)	0.015*
T3 + T4	33	9 (27)	24 (73)	
N-regional lymph node				
N (−)	56	25 (45)	31 (55)	0.919
N (+)	39	17 (44)	22 (56)	
Stage				
I	6	3 (50)	3 (50)	0.235
II	36	18 (50)	18 (50)	
III	17	8 (47)	9 (53)	
IV	36	13 (36)	23 (64)	
Histopathologic type				
Well	60	26 (43)	34 (57)	0.803
Moderately	31	14 (45)	17 (55)	
Poorly	4	2 (50)	2 (50)	
Tumor site				
Gingiva	28	9 (32)	19 (68)	0.456
Tongue	51	25 (49)	26 (51)	
Buccal mucosa	9	4 (44)	5 (56)	
Oral floor	7	4 (57)	3 (43)	

*p < 0.05.

### Establishment of CDCA3 knockdown cells

**Figure 3:**
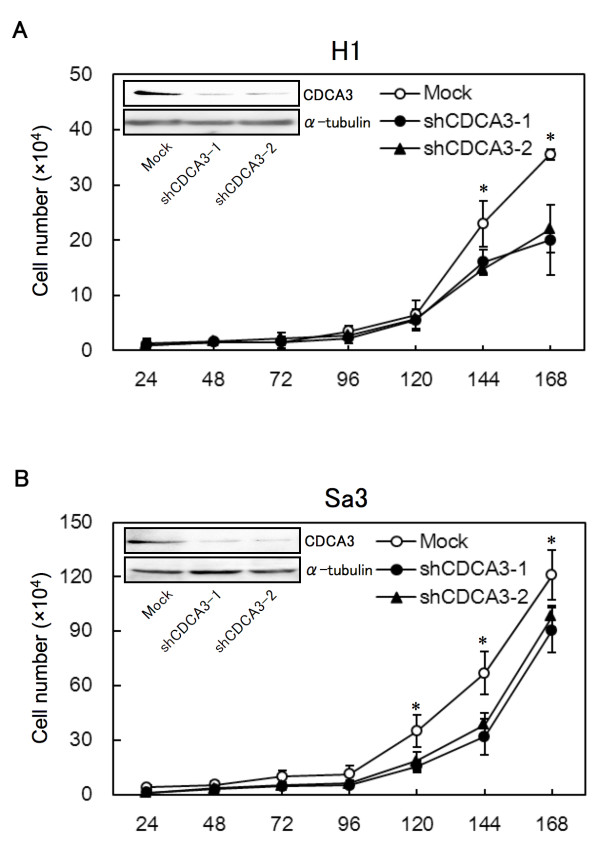
**Proliferation of shCDCA3-transfected H1 (A) and Sa3 (B) cells.** The shCDCA3-transfected H1 and Sa3 cells show a significant (*p < 0.05, Mann-Whitney’s *U* test) decrease in cellular growth compared with mock-transfected cells. The results are expressed as the means ± SEM of values from three assays. Representative Western blot data shows that CDCA3 proteins are markedly down-regulated in shCDCA3-transfected H1 (**A**) and Sa3 (**B**) cells

OSCC-derived cells (H1 and Sa3) transfected with CDCA3 shRNA (shCDCA3) and the control shRNA (mock) plasmid were cloned. qRT-PCR and Western blot analyses were performed to assess the efficiency of CDCA3 knockdown. *CDCA3* mRNA expression in shCDCA3-transfected cells was significantly (*p < 0.05) lower than in mock-transfected cells (Additional file 1A and B). CDCA3 protein levels in shCDCA3-transfected H1 (Figure 3A) and Sa3 (Figure 3B) cells also decreased markedly compared with mock-transfected cells. The densitometric CDCA3 protein levels in shCDCA3-transfected cells decreased significantly (*p < 0.05) compared with the levels in the mock-transfected cells (Additional file 1C and D). The CDCA3 protein expression levels were consistent with the mRNA levels in the transfectants.

### Reduced cellular growth in CDCA3 knockdown cells

To investigate the antiproliferative effects in shCDCA3-transfected cells, cellular growth was monitored for 168 hr. The shCDCA3-transfected H1 (Figure 3A) and Sa3 (Figure 3B) cells showed a significant decrease in cellular growth compared with mock-transfected cells (*p < 0.05).

### Knockdown of CDCA3 promotes cell-cycle arrest with cell-cycle regulators

**Figure 4:**
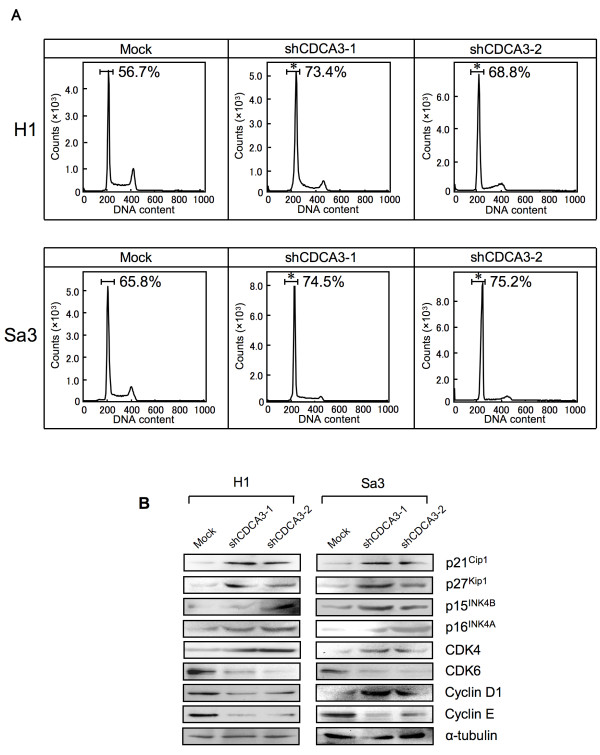
**Flow cytometric determination of DNA content in shCDCA3- and mock-transfected cells. A**) Representative FACS analysis shows that the number of cells in the G1 phase is significantly (*p < 0.05, Mann-Whitney’s *U* test) increased in shCDCA3-transfected H1 and Sa3 cells. **B**) Western blot analysis of CDKIs (p21^Cip1^ p27^Kip1^, p15^INK4B^, and p16^INK4A^), CDK4, CDK6, Cyclin D1, and Cyclin E shows up-regulation of p21^Cip1^, p27^Kip1^, p15^INK4B^, p16^INK4A^, CDK4, and Cyclin D1, and down-regulation of CDK6 and Cyclin E in the CDCA3 knockdown cells

To investigate the mechanism by which down-regulated CDCA3 is related to cell-cycle progression, we performed fluorescence-activated cell sorting (FACS) analysis of shCDCA3-transfected cells. A representative FACS analysis of shCDCA3- and mock-transfected cells is shown in Figure 4A. The percentage of the G1 phase in shCDCA3-transfected cells was significantly (*p < 0.05) higher than in mock-transfected cells. To identify the mechanism by which down-regulated CDCA3 blocks G1 progression, we assessed the protein expression level of cyclin-dependent kinase inhibitors (CDKIs) (p21^Cip1^, p27^Kip1^, p15 ^INK4B^, p16^INK4A^), CDK4, CDK6, Cyclin D1, and Cyclin E (Figure 4B, Additional file 2A, and B). The protein expression data showed up-regulation of p21^Cip1^, p27^Kip1^, p15^INK4B^, p16^INK4A^, CDK4, Cyclin D1, and down-regulation of CDK6 and Cyclin E in the CDCA3 knockdown cells. In addition, to investigate mRNA expression of *Wee1*, a downstream molecule of CDCA3, we performed qRT-PCR analysis using six OSCC-derived cell lines (HSC-2, HSC-3, HSC-4, Ca9-22, H1, and Sa3) and HNOKs. *Wee1* mRNA was significantly (*p < 0.05) down-regulated in all OSCC-derived cell lines compared with the HNOKs (Additional file 3). We also measured *Wee1* expression in CDCA3 knockdown cells. The qRT-PCR data showed that down-regulation of CDCA3 induced a significant (*p < 0.05) increase of *Wee1* mRNA levels compared with mock-transfected cells (Additional file 4A and B).

## Discussion

Our previous microarray data showed significant up-regulation of *CDCA3* in OSCC-derived cell lines [15]. The current study also showed for the first time significant up-regulation of CDCA3 in OSCC-derived cell lines and primary OSCCs compared with the matched normal counterparts. Moreover, CDCA3 protein expression in OPLs was significantly lower than in OSCCs, whereas no significant difference in protein expression was seen between OPLs and normal oral tissues. There was no malignant transformation of OPLs after resection. Low expression of CDCA3 might prevent undergoing malignant transformation of OPLs. In addition, CDCA3 protein expression levels in primary OSCCs were correlated with tumor size (Table 1). These findings indicated that overexpression of CDCA3 might be linked to human oral carcinogenesis and has an important role in OSCC development and progression.

Several cell-cycle regulator proteins are modulated by the SCF complex. To activate the SCF complex, Cul1, a component in the SCF complex, is conjugated with CDCA3 [10]. However, there has been no direct evidence showing that CDCA3 conjugation is required for cell-cycle progression. To determine whether CDCA3 function is relevant to OSCC progression, we performed the shCDCA3 experiment and found that cellular proliferation decreased significantly as a result of cell-cycle arrest at the G1 phase in the CDCA3 knockdown cells with up-regulation of p21^Cip1^, p27^Kip1^, p15 ^INK4B^, and p16^INK4A^ and down-regulation of CDK6 and Cyclin E. These results were consistent with the observations that cell-cycle progression is negatively controlled by CDKIs, such as p21^Cip1^, p27^Kip1^, p57^Kip2^, and the INK4 families (p15^INK4B^, p16^INK4A^, p18^INK4C^, and p19^INK4D^), which are involved in cell-cycle arrest at the G1 phase and have several functions as tumor suppressor genes [7], and that up-regulation of p21^Cip1^ and/or p27^Kip1^ causes growth inhibition in various cancer models [27-29]. The INK4 families can bind to CDK4 and/or to CDK6 and inhibit the catalytic activity of the CDK/Cyclin D complex [30-33]. Cyclin D1, Cyclin E, CDK4, and CDK6 are also critical regulators of G1 progression and G1-S transition [34]. Inhibition of Cyclin D1, Cyclin E, and CDK4 activation blocks G1-S transition in the cell cycle [34-37]. Protein expression in the INK4 families (p15^INK4B^ and p16^INK4A^) was up-regulated in the CDCA3 knockdown cells, whereas the protein expressions of CDK4 and Cyclin D1 were unchanging or increased, suggesting that up-regulation of the INK4 families might suppress the CDK4/Cyclin D1 complex activity. Cyclin D1 is degraded in ubiquitin-proteosome system via the SCF complex. The reason why CDK4/CyclinD1 protein expression were increased in the CDCA3 knockdown cells is considered for inactivation of the SCF complex. We therefore speculated that CDCA3 knockdown leads to impaired activation of the SCF complex, and consistent with that, we found up-regulation of not only the Cip/Kip families (p21^Cip1^ and p27^Kip1^) but also the INK4 families (p15^INK4B^, p16^INK4A^) leading to cell-cycle arrest at the G1 phase in the CDCA3 knockdown cells.

CDCA3 is associated with Wee1 in a phosphospecific manner, and phosphorylation of Wee1 is regulated during the cell cycle [10]. Since Wee1 inactivates CDK1 and Cyclin B during the S and G2 phases, its activity must be down-regulated for mitotic progression to occur [11,38-41]. Wee1 is a key player that serves as a mitotic inhibitor in the intricate network of kinases and phosphatases that regulate the G2 switchboard [41]. The *Wee1* gene has been reported to be underexpressed in colon cancer and non-small cell lung cancer [36,42]. In the current study, we evaluated *Wee1* mRNA expression status in OSCC-derived cell lines. *Wee1* mRNA was significantly (Additional file 2, *p < 0.05) down-regulated in all cell lines compared with the control. *Wee1* mRNA expression then was analyzed in shCDCA3-transfected cells and mock-transfected cells. Wee1 expression was significantly (Additional file 4A and B, *p < 0.05) up-regulated in CDCA3 knockdown cells compared with the control cells; however, CDCA3 knockdown cells stop cell-cycle progression at the G1 phase. These data suggested that G2 arrest was avoided in CDCA3 knockdown cells through activation of cdc25, the counterpart of Wee1, which is the switch for mitosis. Taken together, it is noteworthy that Wee1 expression originally decrease in the H1 and Sa3 cells used for transfection of shCDCA3, indicating that CDCA3 plays a role not only during the G2 phase by mediating degradation of Wee1 but also the G1 phase by mediating degradation of CDKIs in OSCC progression.

## Conclusion

Our results showed that during oral carcinogenesis overexpression of CDCA3 occurs frequently and that it might be closely associated with progression of OSCCs by preventing cessation of cell-cycle progression at the G1 phase, leading to decreased expression of CDKIs. Further studies to identify the interaction between CDCA3 and SCF and additional substrates for cell division cycle genes and to determine how CDCA3 is dysregulated in various cancers and the functional role of CDCA3 during oral carcinogenesis might reveal novel mechanisms for cell-cycle regulation.

## Abbreviations

OSCC, Oral squamous cell carcinoma; HNOKs, Human normal oral keratinocytes; qRT-PCR, Real-time quantitative reverse transcriptase-polymerase chain reaction; IHC, Immunohistochemistry.

## Competing interests

The authors declare that they have no competing interests.

## Authors’ contributions

Conceived and designed the experiments: FU KU AK HTanzawa HB. Performed the experiments: FU AK HTakatori. Analyzed the data: FU AK HTakatori. Contributed reagents/materials/analysis tools: FU KU AK HTakatori YS KO MS HTanzawa HB. Wrote the paper: FU KU AK HTanzawa HB. All authors read and approved the final manuscript.

## Pre-publication history

The pre-publication history for this paper can be accessed here:

http://www.biomedcentral.com/1471-2407/12/321/prepub

## Supplementary Material

Additional file 1 **mRNA and protein expression in shCDCA3-transfected cells using qRT-PCR and Western blot analyses. A, B**) qRT-PCR shows that *CDCA3* is down-regulated in shCDCA3-transfected cells compared with the mock-transfected cells (*p < 0.05, Mann-Whitney’s *U* test). Data are expressed as the means ± SEM of triplicate results. **C, D**) The densitometric CDCA3 protein levels in shCDCA3- and mock-transfected cells show that CDCA3 protein is markedly decreased in shCDCA3-transfected cells compared with mock-transfected cells (*p < 0.05, Mann-Whitney’s *U* test). Click here for file

Additional file 2 **Quantification of CDKIs (p21**^**Cip1**^**, p27**^**Kip1**^**, p15**^**INK4B**^**, and p16**^**INK4A**^**), CDK4, CDK6, Cyclin D1, and Cyclin E protein expression in shCDCA3- and mock-transfected cells.** A, B) Western blot analysis shows up-regulation of p21^Cip1^, p27^Kip1^, p15^INK4B^, p16^INK4A^, CDK4, and Cyclin D1, and down-regulation of CDK6 and Cyclin E in the CDCA3 knockdown cells. Click here for file

Additional file 3 **Quantification of*Wee1*mRNA expression in OSCC-derived cell lines by qRT-PCR analysis.** Significant down-regulation of *Wee1* mRNA is seen in six OSCC-derived cell lines compared with the HNOKs (*p < 0.05, Mann-Whitney’s *U* test). Data are expressed as the means ± SEM of triplicate results. Click here for file

Additional file 4 **Quantification of*Wee1*mRNA expression in shCDCA3- and mock-transfected cells by qRT-PCR analysis.** Significant up-regulation of *Wee1* mRNA is seen in shCDCA3-transfected H1 (**A**) and Sa3 (**B**) cells compared with the mock-transfected cells (*p < 0.05, Mann-Whitney’s *U* test). Data are expressed as the means ± SEM of triplicate results. Click here for file
