# Supplementary figures and images for: Overexpression of cell cycle regulator CDCA3 promotes oral cancer progression by enhancing cell proliferation with prevention of G1 phase arrest
Source: BMC Cancer. 2012 Jul 28;12:321. doi: 10.1186/1471-2407-12-321 (PMC3418557; doi:10.1186/1471-2407-12-321)

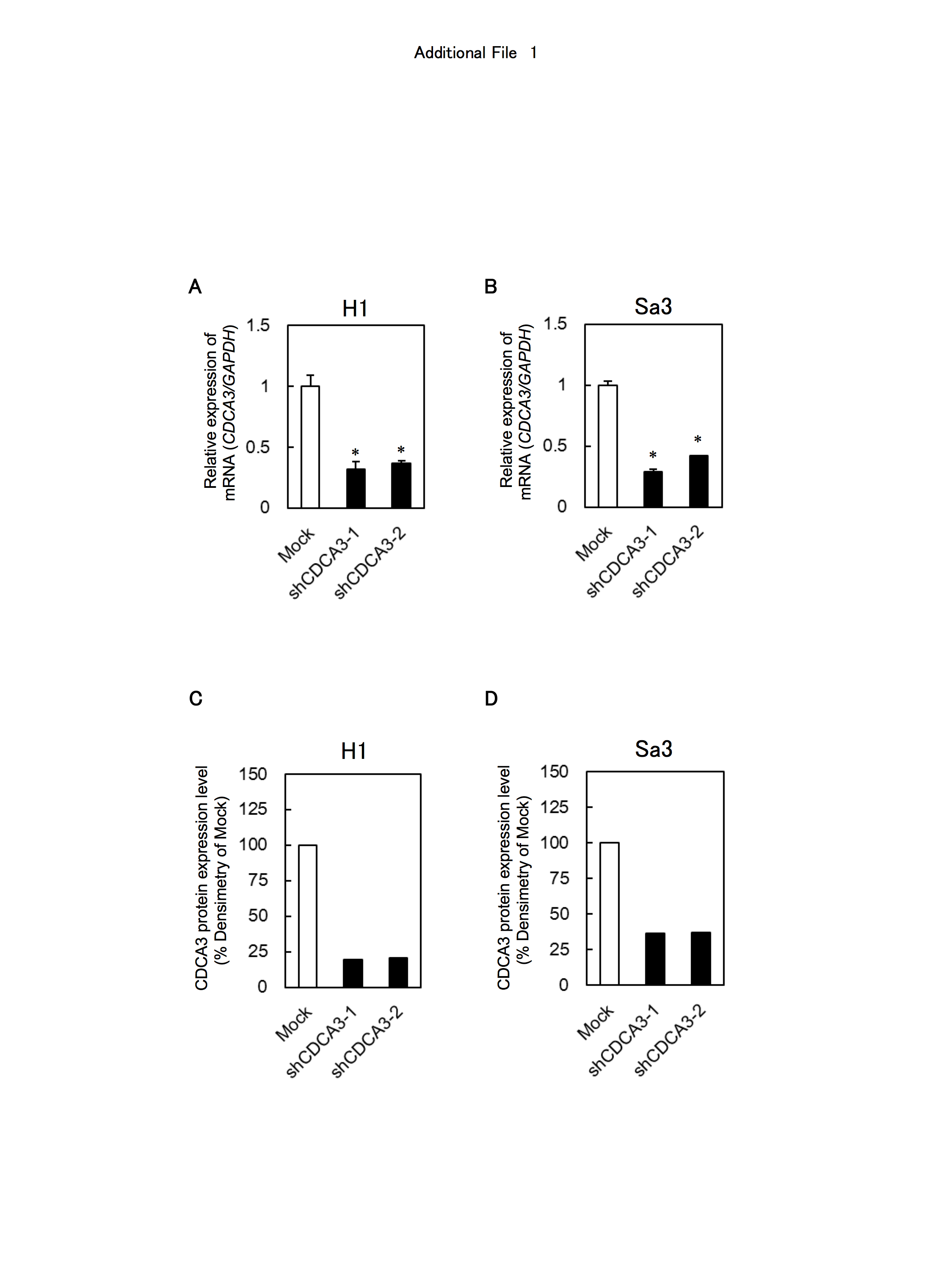

Supplement: Additional file 1 — mRNA and protein expression in shCDCA3-transfected cells using qRT-PCR and Western blot analyses. A, B) qRT-PCR shows that CDCA3 is down-regulated in shCDCA3-transfected cells compared with the mock-transfected cells (*p < 0.05, Mann-Whitney’s U test). Data are expressed as the means ± SEM of triplicate results. C, D) The densitometric CDCA3 protein levels in shCDCA3- and mock-transfected cells show that CDCA3 protein is markedly decreased in shCDCA3-transfected cells compared with mock-transfected cells (*p < 0.05, Mann-Whitney’s U test). [file 1471-2407-12-321-S1.tiff]

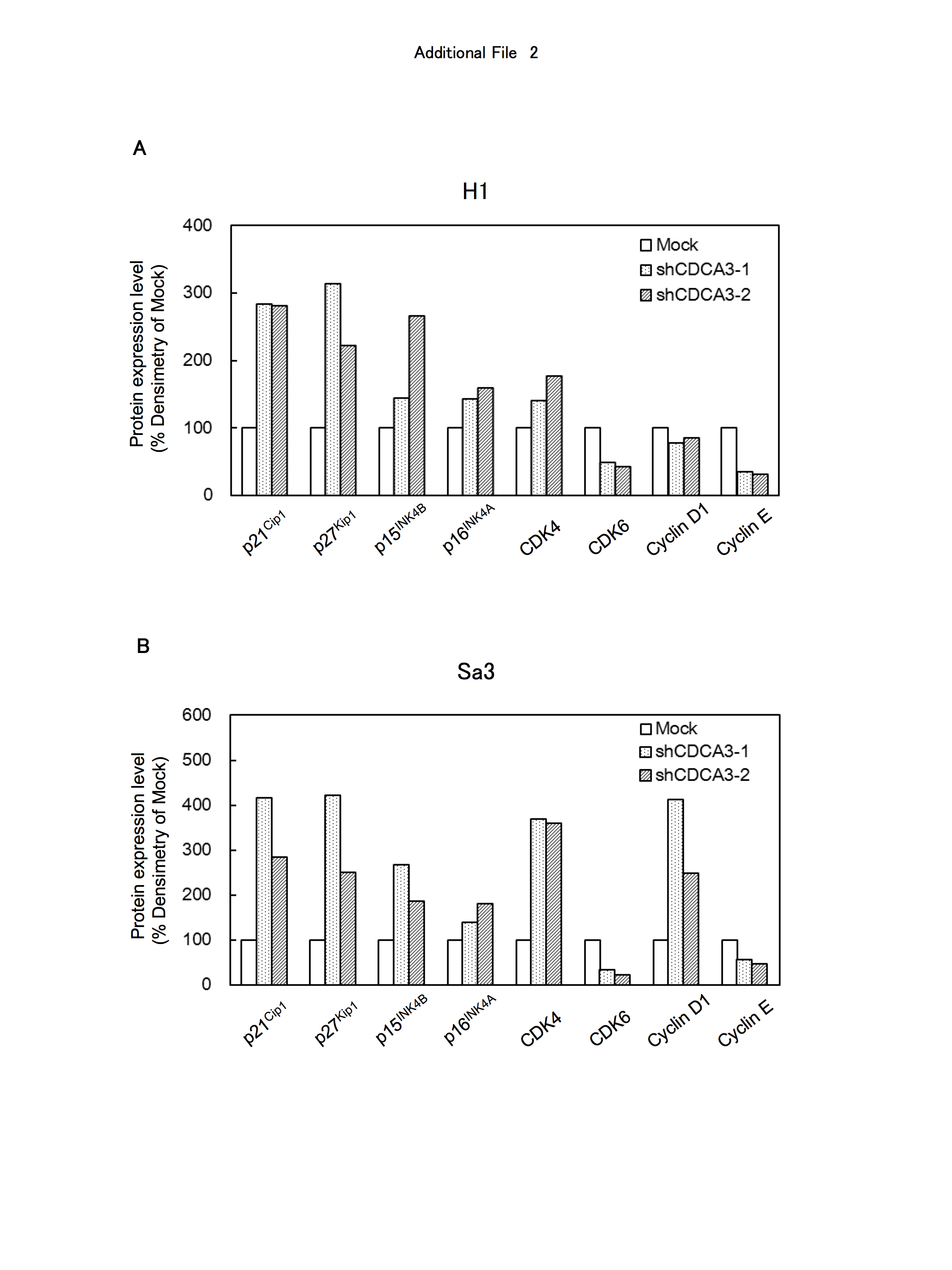

Supplement: Additional file 2 — Quantification of CDKIs (p21Cip1, p27Kip1, p15INK4B, and p16INK4A), CDK4, CDK6, Cyclin D1, and Cyclin E protein expression in shCDCA3- and mock-transfected cells. A, B) Western blot analysis shows up-regulation of p21Cip1, p27Kip1, p15INK4B, p16INK4A, CDK4, and Cyclin D1, and down-regulation of CDK6 and Cyclin E in the CDCA3 knockdown cells. [file 1471-2407-12-321-S2.tiff]

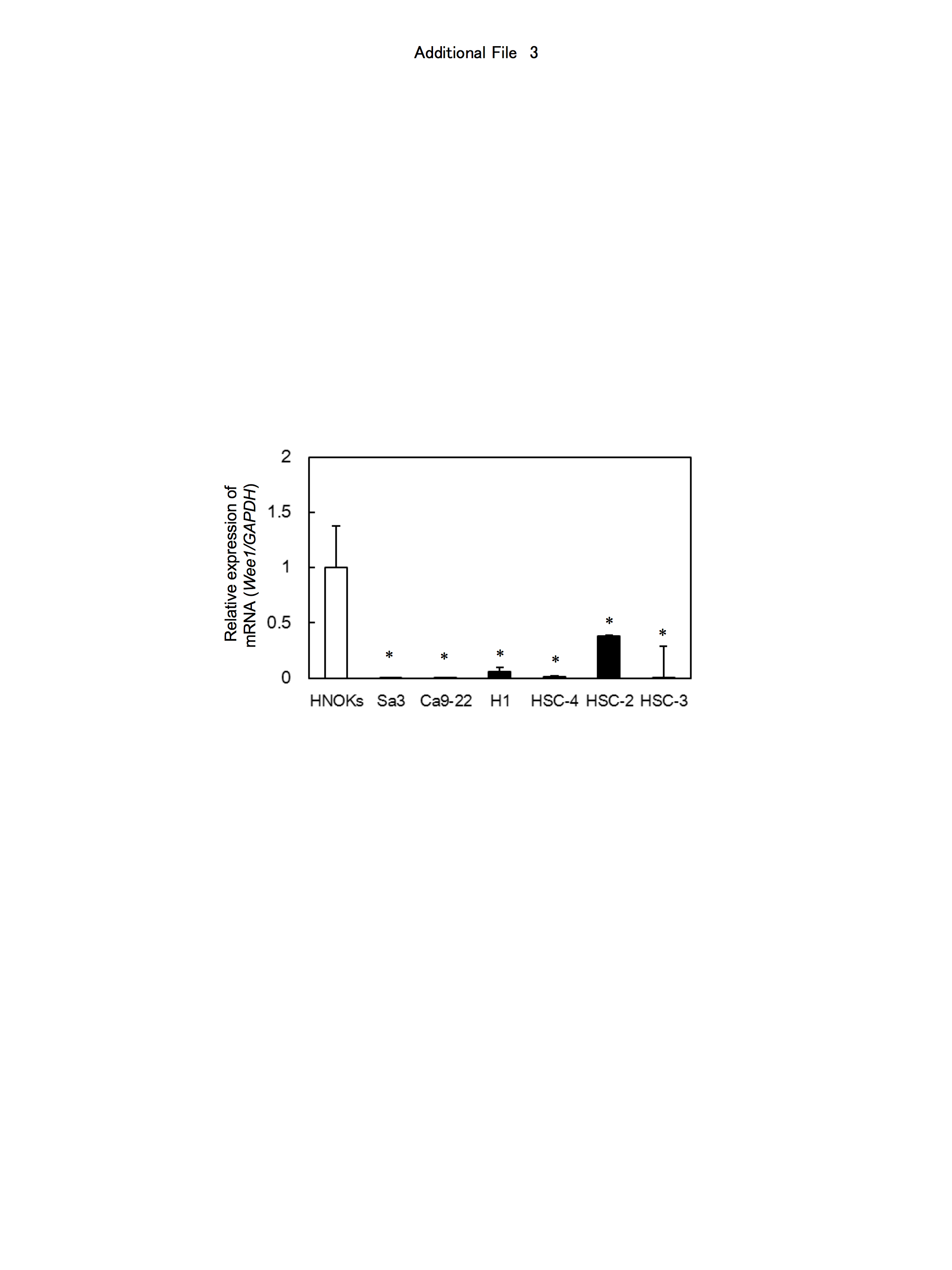

Supplement: Additional file 3 — Quantification ofWee1mRNA expression in OSCC-derived cell lines by qRT-PCR analysis. Significant down-regulation of Wee1 mRNA is seen in six OSCC-derived cell lines compared with the HNOKs (*p < 0.05, Mann-Whitney’s U test). Data are expressed as the means ± SEM of triplicate results. [file 1471-2407-12-321-S3.tiff]

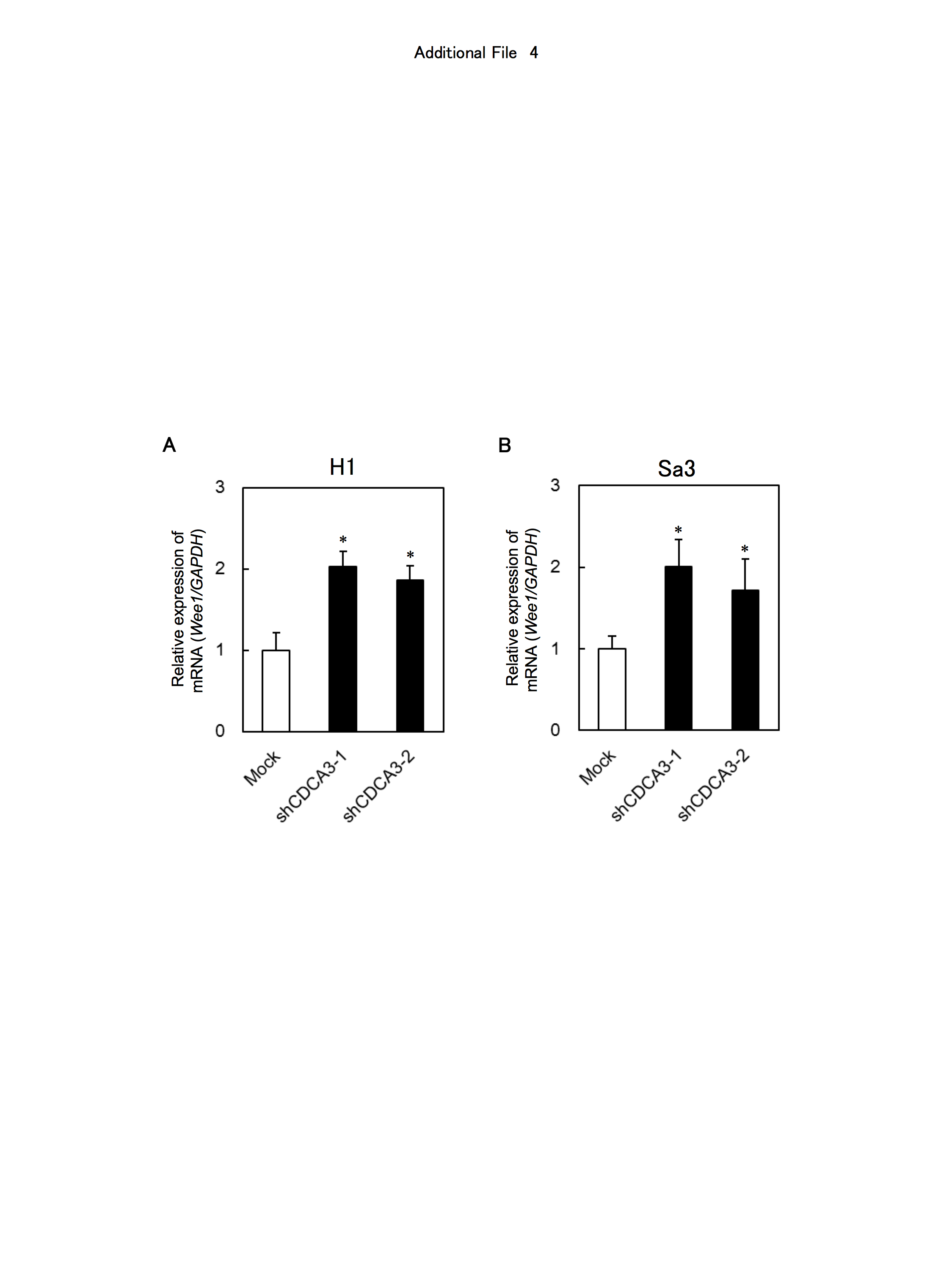

Supplement: Additional file 4 — Quantification ofWee1mRNA expression in shCDCA3- and mock-transfected cells by qRT-PCR analysis. Significant up-regulation of Wee1 mRNA is seen in shCDCA3-transfected H1 (A) and Sa3 (B) cells compared with the mock-transfected cells (*p < 0.05, Mann-Whitney’s U test). Data are expressed as the means ± SEM of triplicate results. [file 1471-2407-12-321-S4.tiff]
